# Effectiveness of Individualized Autovaccine Immunotherapy in Women With Recurrent Urinary Tract Infections: A Prospective Cohort Study

**DOI:** 10.1093/ofid/ofag315

**Published:** 2026-05-21

**Authors:** Vicens Diaz-Brito, Montserrat Sanmartí, Araceli González-Cuevas, Antonio Cárdenas, Maria Eugenia Guerrero, Anna Capella, Eva Martínez-Franco, Montserrat Margarit, Anna Agud, Juan Maria Bastaros, Francisco Medina, Encarna Moreno, María Carmen Álvarez, Erika Esteve-Palau

**Affiliations:** Department of Infectious Diseases, Parc Sanitari Sant Joan de Deu, Sant Boi (Barcelona), Spain; Sant Joan de Deu Research Institute, Esplugues (Barcelona), Spain; Department of Infectious Diseases, Parc Sanitari Sant Joan de Deu, Sant Boi (Barcelona), Spain; Sant Joan de Deu Research Institute, Esplugues (Barcelona), Spain; Sant Joan de Deu Research Institute, Esplugues (Barcelona), Spain; Department of Microbiology, Parc Sanitari Sant Joan de Deu, Sant Boi (Barcelona), Spain; Department of Infectious Diseases, Parc Sanitari Sant Joan de Deu, Sant Boi (Barcelona), Spain; Sant Joan de Deu Research Institute, Esplugues (Barcelona), Spain; Sant Joan de Deu Research Institute, Esplugues (Barcelona), Spain; Department of Microbiology, Parc Sanitari Sant Joan de Deu, Sant Boi (Barcelona), Spain; Sant Joan de Deu Research Institute, Esplugues (Barcelona), Spain; Department of Pharmacy, Parc Sanitari Sant Joan de Deu, Sant Boi (Barcelona), Spain; Sant Joan de Deu Research Institute, Esplugues (Barcelona), Spain; Department of Gynecology, Parc Sanitari Sant Joan de Deu, Sant Boi (Barcelona), Spain; Sant Joan de Deu Research Institute, Esplugues (Barcelona), Spain; Department of Gynecology, Parc Sanitari Sant Joan de Deu, Sant Boi (Barcelona), Spain; Sant Joan de Deu Research Institute, Esplugues (Barcelona), Spain; Department of Urology, Parc Sanitari Sant Joan de Deu, Sant Boi (Barcelona), Spain; Sant Joan de Deu Research Institute, Esplugues (Barcelona), Spain; Department of Urology, Parc Sanitari Sant Joan de Deu, Sant Boi (Barcelona), Spain; Department of Infectious Diseases, Parc Sanitari Sant Joan de Deu, Sant Boi (Barcelona), Spain; Sant Joan de Deu Research Institute, Esplugues (Barcelona), Spain; Department of Infectious Diseases, Parc Sanitari Sant Joan de Deu, Sant Boi (Barcelona), Spain; Sant Joan de Deu Research Institute, Esplugues (Barcelona), Spain; Department of Infectious Diseases, Parc Sanitari Sant Joan de Deu, Sant Boi (Barcelona), Spain; Sant Joan de Deu Research Institute, Esplugues (Barcelona), Spain; Department of Infectious Diseases, Parc Sanitari Sant Joan de Deu, Sant Boi (Barcelona), Spain; Sant Joan de Deu Research Institute, Esplugues (Barcelona), Spain

**Keywords:** antibiotic resistance, autovaccine, immunotherapy, nonantibiotic prophylaxis, recurrent urinary tract infection (rUTI)

## Abstract

**Background:**

Recurrent urinary tract infections (rUTIs) disproportionately affect older women and are frequently driven by nonmodifiable risk factors (nmRFs). This study evaluated the effectiveness, safety, and patient-reported outcomes (PROs) of personalized sublingual immunoprophylaxis in women with rUTIs.

**Methods:**

Prospective, single-center, noncomparative observational cohort study. Women with rUTIs received a Uromune® autovaccine prepared from inactivated strains isolated from each patient's urine culture (UC). Recurrence episodes and UC results were recorded at baseline and at 3, 9, and 12 months. PROs (daily activity, QAct; emotional distress, QEm) were assessed in a predefined substudy. Longitudinal mixed-effects and population-averaged models evaluated change over time and explored baseline risk factors as effect modifiers.

**Results:**

Of 119 screened women, 100 were enrolled (median [IQR] age: 73 [61–80] years); most presented multiple nmRFs. Median (IQR) rUTI episodes decreased from 4 (3–5) at baseline to 0 (0–2) at 3 months and remained 1 (0–2) and 1 (0–3) at 9 and 12 months (all *P* < .001). Approximately 40% were UTI-free in each follow-up interval. UC negativity was 50.7% at 3 months, decreased at 9 months (25.9%), and increased again at 12 months (45.5%), remaining higher than baseline. QEm improved significantly at 3 and 12 months; QAct showed a trend toward improvement. Vaccine-related adverse events were uncommon (6%) and mild.

**Conclusions:**

Individualized sublingual immunoprophylaxis was safe and associated with sustained reductions in rUTI episodes, improved microbiological findings, and better emotional well-being, suggesting a potential role as a nonantibiotic preventive strategy. These observational findings are hypothesis-generating and warrant confirmation in controlled studies.

## Teaser

Individualized sublingual immunoprophylaxis was safe and associated with fewer recurrent UTI episodes, a higher proportion of negative urine cultures, and improved emotional well-being over 12 months. Similar patterns were observed across age groups and risk-factor profiles, suggesting a potential personalized nonantibiotic preventive approach.

## Graphical Abstract

**Graphical Abstract:**
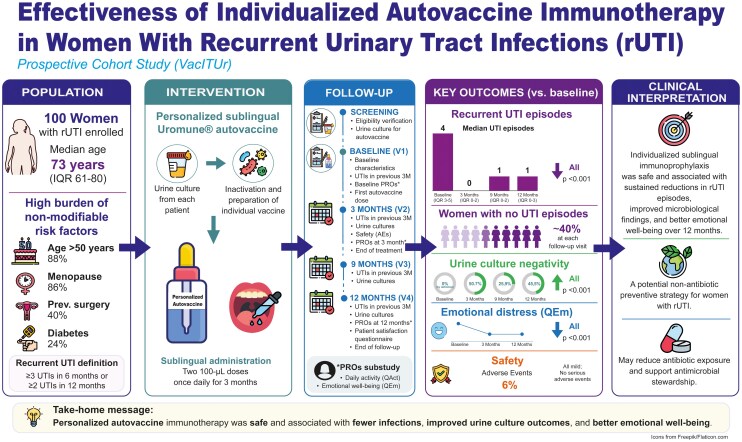

Urinary tract infections (UTIS) are among the most prevalent bacterial infections worldwide, with more than 400 million cases and over 236 000 UTI-related deaths reported globally [1, 2]. They also impose a substantial healthcare and economic burden, accounting for millions of healthcare visits annually and considerable costs [3–7].

Women are disproportionately affected, with recurrence rates increasing with age, particularly after menopause. Over half experience at least one UTI during their lifetime, and up to 30% develop recurrent UTIs (rUTIs), with 3%–5% meeting criteria for high-frequency recurrence [7, 8]. International guidelines define rUTI as ≥3 UTIs per year or ≥2 in 6 months [9–11]. Beyond the clinical burden, rUTIs impair quality of life and psychological well-being [12, 13].

The pathogenesis of UTIs reflects a complex interplay between bacterial virulence, inoculum size, and host factors, with uropathogen colonization as the initial step toward infection [14, 15]. *Escherichia coli* accounts for 75%–90% of cases [16], owing to its ability to adhere to and invade uroepithelial cells [17]. Other uropathogens include *Klebsiella pneumoniae*, *Enterococcus spp.*, *Proteus mirabilis*, and *Staphylococcus saprophyticus* [11]. Female-specific anatomical features, factors facilitating bacterial ascent, and conditions promoting colonization or reducing urinary flow constitute key risk factors, many of them nonmodifiable and prevalent in postmenopausal women [14, 18, 19].

Antibiotics remain the cornerstone of UTI treatment and prophylaxis, accounting for 25%–40% of antibiotic prescriptions [20, 21]. However, prolonged exposure is associated with adverse effects, microbiota disruption, and antimicrobial resistance (AMR) [22, 23]. Rising AMR leads to higher treatment failure rates, escalating healthcare costs, and greater use of broad-spectrum agents [24, 25]. In Europe, more than 500 000 antibiotic-resistant UTIs are reported annually, with estimated costs of €11.7 billion [26, 27]. These challenges underscore the need for effective nonantimicrobial prophylactic (nAMP) strategies to reduce antibiotic exposure and improve long-term outcomes [28].

Current guidelines support a stepwise approach: risk-factor counseling, nAMP measures, and antimicrobial prophylaxis only when necessary [9, 11]. Among nAMP options—including increased fluid intake [29], methenamine hippurate [30–32], topical estrogen in postmenopausal women [33–35], intravesical glycosaminoglycans [36–38], and nutraceuticals (probiotics, D-mannose, and cranberry) [39–46]—immunoactive prophylaxis has emerged as a promising strategy, reducing recurrence rates and antibiotic use [47, 48].

Several studies have shown that MV140-Uromune®—a sublingual polybacterial vaccine composed of inactivated *E. coli, K. pneumoniae, E. faecalis,* and *P. vulgaris*—significantly reduces rUTI episodes and has a favorable safety profile [22, 47–59]. Individualized autovaccines, based on patient-specific uropathogens, may provide closer antigenic matching than fixed-composition vaccines and enhance mucosal immune responses through innate and adaptive activation, potentially reducing colonization and bacterial ascent [49]. In Spain, regulatory changes in 2018 led to Uromune® being manufactured exclusively as an individualized autovaccine, increasing its use and representing a distinct approach compared with standardized vaccines [60]. However, evidence on the effectiveness of personalized autovaccines in routine clinical practice remains limited [60–63].

The present VacITUr study evaluated clinical and microbiological outcomes, safety, and health-related quality-of-life (HRQoL) of individualized Uromune® autovaccine prophylaxis in high-risk women with rUTIs in a real-world setting.

## METHODS

### Study Design and Setting

A prospective, single-center, noncomparative observational cohort study was conducted between January 2019 and December 2023 at the multidisciplinary outpatient rUTI clinic of Parc Sanitari Sant Joan de Déu (Sant Boi de Llobregat, Barcelona, Spain). The study evaluated clinical and microbiological outcomes, safety, and patient-reported outcomes (PROs) of once-daily individualized sublingual Uromune® autovaccine administered for 3 months in women with rUTIs.

The study adhered to the Declaration of Helsinki, was approved by the local Institutional Review Board, and all participants provided written informed consent. Data were anonymized before analysis.

### Study Participants

Inclusion criteria for this study were:

- Women ≥18 years.
- Diagnosed with rUTI, defined as ≥2 episodes within 6 months or ≥3 within 1 year [9–11].
- No prior bacterial immunotherapy.
- Ability to provide informed consent.

Exclusion criteria were:

- Refusal or inability to understand the participant information sheet or provide informed consent.
- Known allergy to any vaccine component.
- Concomitant rUTI prophylaxis (eg, antibiotic therapy or nutraceutical preventive treatments), excluded to minimize confounding.
- Presence of urinary catheters.
- Moderate to severe cognitive impairment.

### Endpoints

#### Primary Endpoint

- Change in rUTI episode frequency from baseline to the end of treatment (3 months).

#### Secondary Endpoints

- Change in rUTI episode frequency from baseline to 9 and 12 months.
- Proportion of negative urine cultures (UC; ie, bacteriological clearance) at 3, 9, and 12 months.
- Association of nonmodifiable risk factors (nmRFs) with clinical (recurrence episodes) and bacteriological (UC negativity) outcomes.
- Incidence of vaccine-related adverse events (AEs) through 3 months of treatment.
- Analysis of PROs, including HRQoL (daily activities and emotions questionnaires) and patient satisfaction, assessed in a predefined exploratory substudy including the first one-third of consecutively enrolled patients without cognitive impairment.

### Vaccine Preparation and Administration

Only customized Uromune® (Q Pharma/Inmunotek Laboratories, Spain) autovaccines were used. For each patient, uropathogens were isolated from her routine UCs in the hospital microbiology laboratory, stored frozen, and subsequently transferred onto transport swabs in Amies medium for shipment at room temperature to Q Pharma/Inmunotek Laboratories (Alicante, Spain) for autovaccine preparation according to the manufacturer's standard procedures. Bacterial isolates were heat-inactivated and adjusted to 300 formazin turbidity units (≈10^9^ bacteria/mL) in a glycerinated whole-cell suspension containing sodium chloride, artificial pineapple flavoring, and water for sublingual administration [53]. For each patient, a single manufacturing batch was produced and used throughout the 3-month treatment period.

Each autovaccine included only strains isolated from the corresponding patient (monovalent when a single organism was recovered; polybacterial with equal representation when multiple organisms were identified).

Vaccination consisted of two 100-µL sublingual sprays self-administered once daily on an empty stomach for 3 months (≈10^8^ inactivated bacteria per spray), with storage at 2°C–8°C according to the manufacturer's instructions. Acute UTI episodes were treated with antibiotics when clinically indicated. No additional nutraceuticals or antibiotic prophylaxis were permitted.

### Study Procedures and Follow-Up

Participants attended scheduled visits throughout the study. During screening, eligibility criteria were verified, a UC was obtained for vaccine manufacture, and isolated pathogens were recorded.

At the baseline visit (Day 1 of treatment), demographics, rUTI risk factors, prior antibiotic prophylaxis, and UTI episodes in the previous 3 months were recorded, and patients received autovaccine self-administration instructions. Participants enrolled in the PROs substudy also completed baseline HRQoL questionnaires.

**Figure 1.**
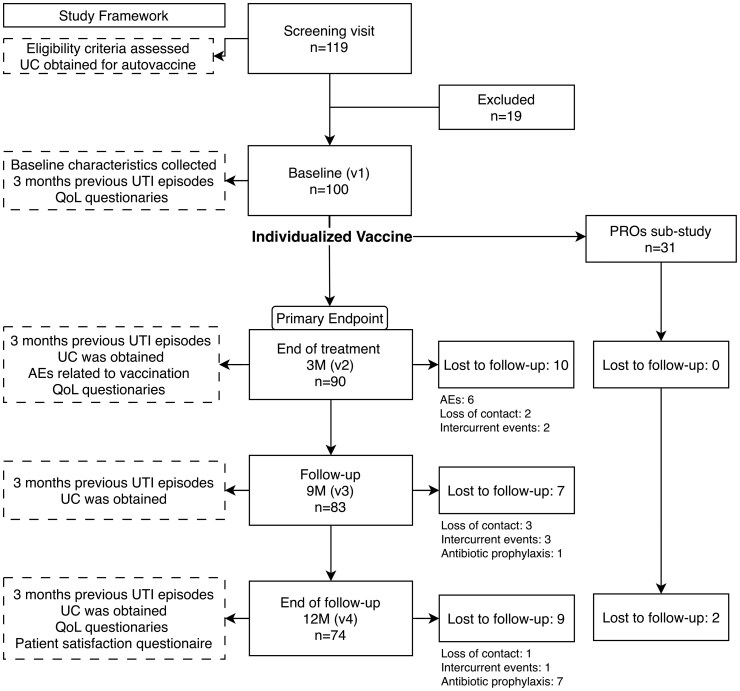
VacITUr study flow diagram. UTI, urinary tract infection; UC, urine culture; AEs, adverse events; 3M, 3 m; 9M, 9 m; 12M, 12 m; V1, visit 1; V2, visit 2; V3, visit 3; V4, visit 4; Intercurrent events, intercurrent non-UTI events; Antibiotic prophylaxis, initiation of antibiotic prophylaxis. Baseline assessments included demographics, rUTI risk factors, and prior use of antibiotic prophylaxis. The patient-reported outcomes (PROs) substudy evaluated daily activity and emotional distress domains of quality of life (QoL), and included a patient satisfaction questionnaire at the end of follow-up.

Follow-up visits occurred at 3, 9, and 12 months. At each visit, UTI episodes were documented, and a UC was obtained. Vaccine-related AEs were evaluated at the 3-month visit. Patients in the PROs substudy completed HRQoL assessments at months 3 and 12, and treatment satisfaction was assessed at the final visit (Figure 1).

Risk factors for rUTI were categorized according to their potential for modification; nmRFs included age ≥50 years, menopause, family history of UTIs, congenital urinary tract anomalies, cognitive impairment, history of premenopausal rUTI, previous urogenital surgery, neurogenic bladder, and diabetes mellitus. Difficult-to-modify factors comprised immunosuppression, fibromyalgia, pelvic-organ prolapse, postvoid residual urine, fecal incontinence, cystocele or rectocele, sexual activity-related rUTI, and recurrent nephrolithiasis. Modifiable factors were urinary incontinence, constipation, and the use of spermicides. These baseline variables were prospectively defined and evaluated as potential effect modifiers in secondary longitudinal analyses.

Participants were instructed to keep a diary to record symptoms and medication use and were advised to seek clinical evaluation whenever possible in the event of suspected infection or AEs.

UTI episodes were defined as a positive UC (≥10^3^ CFU/mL of an accepted uropathogen) accompanied by compatible symptoms requiring antibiotic treatment and improving after therapy. UCs were not systematically required for each rUTI episode, reflecting routine clinical practice. When UC was unavailable or contaminated, UTI episodes were defined as symptoms persisting for more than 3 days without prior treatment and resolving following antimicrobial therapy. AEs were evaluated for severity and causality with respect to vaccination at the 3-month visit.

HRQoL was assessed using adapted versions of the Leicester Impact Scale, which evaluates the impact of rUTIs on daily activities (QAct) and emotional well-being (QEm) [64]. Treatment satisfaction was measured using an adapted Treatment Satisfaction Questionnaire for Medication [65, 66].

Participants were discontinued from the study if they withdrew consent, experienced a common terminology criteria for AEs grade 4 clinical AE deemed related to vaccination, or initiated other rUTI prophylaxis (nutraceuticals or antibiotics).

### Sample Size and Statistical Analysis

Because no prior data were available for personalized autovaccines for rUTI prophylaxis, no formal sample size calculation was feasible. The study was therefore conceived as exploratory, and a target sample of ∼100 women was selected to ensure adequate precision in estimating effect sizes, consistent with previous prospective studies using the generic MV140 formulation. With 100 paired observations and α = .05, the study would have ∼80% power to detect a small within-patient difference (0.28 SD), equivalent to about half a UTI episode.

Categorical endpoints were summarized as counts and percentages, and continuous endpoints as mean (standard deviation, SD) or median (interquartile range, IQR), as applicable.

The primary endpoint was analyzed using the Wilcoxon signed-rank test. Longitudinal changes in rUTI episodes were analyzed using linear mixed-effects models (LMMs) to account for repeated within-patient measurements and incomplete follow-up, with baseline risk factors evaluated as potential effect modifiers. UTI-free status (no episode) was evaluated using McNemar's test for paired comparisons and population-averaged logistic models using generalized estimating equations, with risk factors examined in secondary analyses.

Participants lost to follow-up or initiating prohibited prophylaxis were censored at that time point; subsequent data were treated as missing. Longitudinal models were estimated using all available observations under the assumption that data were missing at random. Safety and satisfaction outcomes were summarized descriptively. PROs were analyzed using nonparametric tests or LMMs according to distribution.

All statistical tests were two-sided with a significance threshold of *P* < .05. Analyses were performed using R version 4.5.

## RESULTS

### Baseline Demographics and Clinical Characteristics

A total of 119 women were screened, of whom 100 met eligibility criteria and were enrolled. Reasons for exclusion appear in Figure 1. The median (IQR) age was 73 (61–80) years, and 31 participants were included in the PROs substudy.

Most women presented multiple risk factors for rUTI, with nonmodifiable factors predominating (Table 1). Risk factors were treated as nonmutually exclusive variables. The most frequent risk factors included age ≥50 years, menopause, urinary incontinence, and previous urogenital surgery. Individual risk-factor distributions are shown in Supplementary Figure A1. Nearly, half of participants (49%) had received prior antibiotic prophylaxis.

**Table 1.** Baseline Demographics and Clinical Characteristics

Baseline Demographics and Clinical Characteristics	*N* = 100
Age (y), median (IQR)	73 (61–80)
Prev. ABX prophylaxis, *n* (%)	49 (49.0)
Total risk factors p/p, median (IQR)	4 (3–6)
nmRF p/p, median (IQR)	3 (2–4)
Age > 50 y, *n* (%)	88 (88.0)
Menopause, *n* (%)	86 (86.0)
Urogenital surgery, *n* (%)	40 (40.0)
Diabetes mellitus, *n* (%)	24 (24.0)
Cognitive impairment, *n* (%)	18 (18.0)
Premenopausal rUTI, *n* (%)	12 (12.0)
Neurogenic bladder, *n* (%)	5 (5.0)
First-degree relative with a history of UTI, *n* (%)	5 (5.0)
UT malformation, *n* (%)	4 (4.0)
Diffi. to modify. RF p/p, median (IQR)	1 (1–2)
Cystocele/rectocele, *n* (%)	30 (30.0)
Fibromyalgia, *n* (%)	27 (27.0)
Postvoid residual urine, *n* (%)	27 (27.0)
Pelvic-organ prolapse, *n* (%)	21 (21.0)
Recurrent nephrolithiasis, *n* (%)	16 (16.0)
Fecal incontinence, *n* (%)	13 (13.0)
Immunosuppression, *n* (%)	8 (8.0)
Sex-related, *n* (%)	7 (7.0)
Modifiable RF p/p, median (IQR)	1 (1–2)
Urinary incontinence, *n* (%)	62 (62.0)
Constipation, *n* (%)	34 (34.0)
Use of spermicides, *n* (%)	0 (0.0)

Abbreviations: Diffi., to modify; IQR, interquartile range; p/p, per patient; nmRF, nonmodifiable risk factor; RF, difficult-to-modify risk factor; Sex-related, sexual activity-related recurrent UTI; UT. Malform, congenital malformations of the urinary tract.

Risk factors were categorized according to their theoretical potential for clinical or behavioral intervention, consistent with guideline-based preventive strategies for rUTI [9–11]. Difficult-to-modify factors refer to conditions potentially amenable to intervention but not readily reversible.

Baseline UC isolates used for autovaccine formulation were predominantly *Enterobacteriaceae* (94 [94.0%]), mainly *E. coli* (67 [67.0%]) and *K. pneumoniae* (20 [20.0%]). Most autovaccines (87 [87.0%]) were monovalent; the full microorganism profile appears in Supplementary Figure A2.

### Primary Endpoint

**Figure 2.**
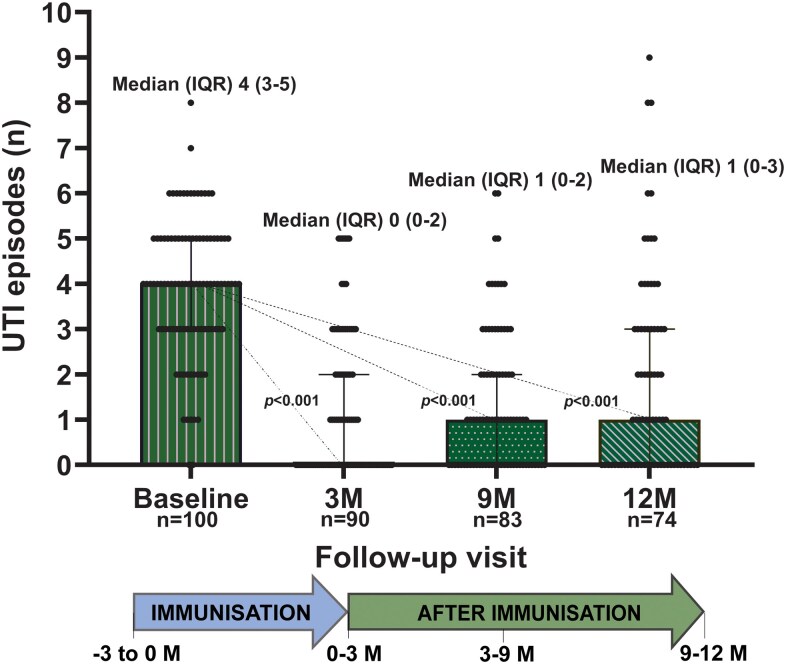
Evolution of rUTI episodes over time following individualized autovaccination. Distribution of rUTI episodes at baseline and at 3-, 9-, and 12-month follow-up visits. Values are shown as medians (IQR) from paired Wilcoxon tests versus baseline.

In the 3 months preceding baseline, patients experienced a median (IQR) of 4 (3–5) rUTI episodes. After the 3-month autovaccine regimen, a statistically significant difference was observed (*P* < .001), with the median (IQR) recurrence falling to 0 (0–2) episodes (Figure 2 and Supplementary Figure A3a and A3b).

### Secondary Endpoints

#### UTI Recurrence During Follow-up

The reduction in rUTI episodes observed at 3 months was maintained throughout follow-up. At 9 and 12 months, median (IQR) episodes were 1 (0–2) and 1 (0–3), respectively, remaining significantly lower than baseline (*P* < .001 for both; Figure 2). Approximately 40% of women remained UTI-free (0 UTI episodes) at each follow-up visit (Supplementary Figure A3a). Overall, 56.2% of rUTI episodes recorded during follow-up were culture-confirmed.

Longitudinal mixed-effects models showed significant reductions at all visits (Supplementary Figure A3b).

#### Bacteriological Clearance

**Figure 3.**
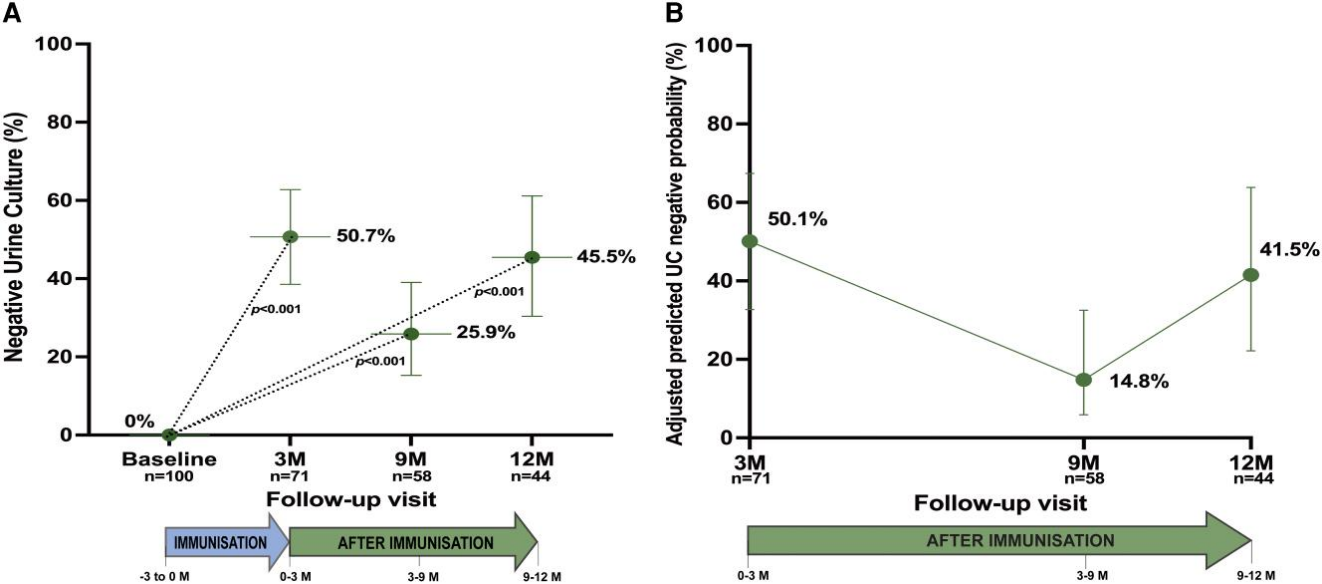
Evolution of bacteriological clearance after individualized immunoprophylaxis. UC, urine culture; M, months. *n* represents patients with evaluable urine cultures at each timepoint. *A*, Observed bacteriological clearance at each visit. Proportion of patients with negative urine cultures at baseline and at 3-, 9-, and 12-month follow-up visits. Differences versus baseline were evaluated using Fisher's exact tests. *B*, Longitudinal model-estimated probability of bacteriological clearance. Adjusted predicted probabilities over time, obtained from a binomial generalized linear mixed-effects model.

Negative UCs were more frequent after immunization, with 50.7% (36/71) of evaluable UCs testing negative at 3 months compared with 0% at baseline (Figure 3*A*). At 9 and 12 months, the proportion of negative cultures was 25.9% (15/58) and 45.5% (20/44), respectively, with all postbaseline values significantly higher than baseline (all *P* < .001).

Model-adjusted analyses, which excluded baseline due to uniform culture positivity, showed a similar pattern of fluctuation over time (Figure 3*B*). The adjusted probability of a negative culture was estimated at 50.1% at 3 months, was 14.8% at 9 months (*P* = .002 vs 3 M), and 41.5% at 12 months (*P* = .500 vs 3 M), indicating variability across visits but sustained differences relative to baseline.

The distribution of bacterial isolates recovered during follow-up resembled that of the autovaccine components, with *E. coli* and *K. pneumoniae* remaining the most frequent (Supplementary Figure A4).

#### Influence of Risk Factors

In mixed-effects models adjusting for age and prior prophylaxis, rUTI episodes were significantly lower at all follow-up visits compared with baseline (Supplementary Table A1). Neither variable showed a consistent modifying effect across time.

**Figure 4.**
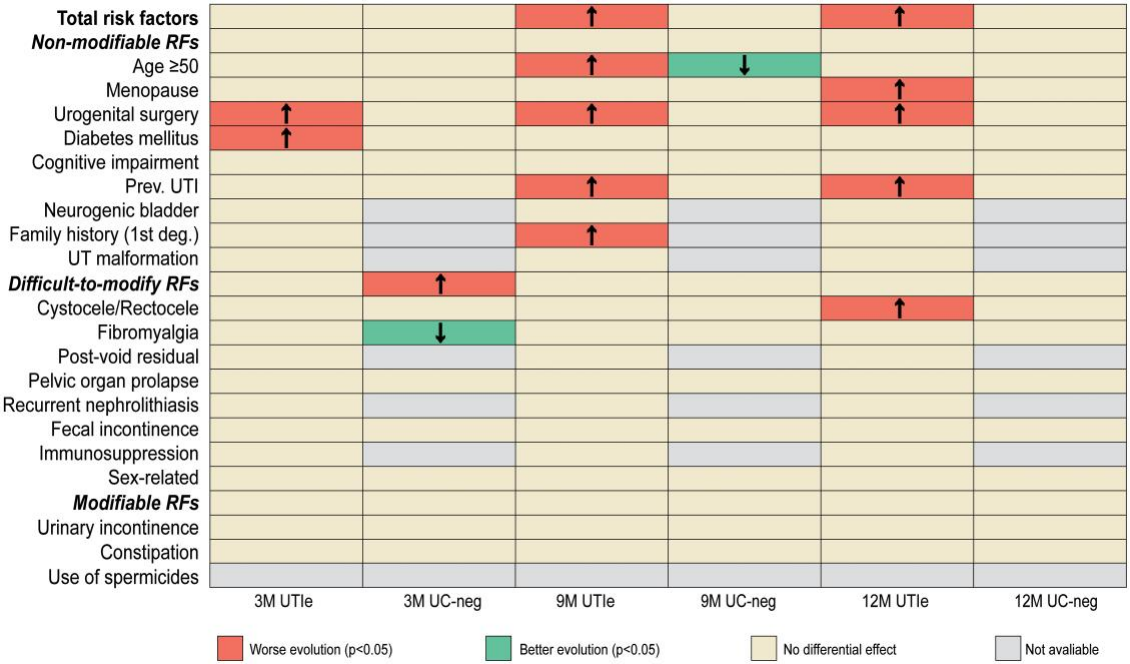
Associations between baseline risk factors and longitudinal changes in UTI recurrence and UC negativity at 3, 9, and 12 month. RFs, risk factors; Prev. UTI, history of premenopausal recurrent urinary tract infection (UTI); family history (first deg.), family history of UTI in first-degree relatives; UT malformation, congenital malformations of the urinary tract; Postvoid residual, postvoid residual urine; Sex-related, sexual activity-related recurrent UTI; M, month; UTIe, UTI episodes; UC-neg, urine culture negativity. Most baseline risk factors showed no consistent modifying effect across visits. Reductions in UTI recurrence were broadly observed across risk-factor categories. Arrows indicate statistically significant differences (*P* < .05) in longitudinal changes relative to baseline. Gray cells denote unavailable estimates.

Fewer recurrences were observed across all risk-factor categories—nmRFs, difficult-to-modify, and modifiable—with no sustained association between individual or grouped risk factors and clinical or bacteriological outcomes (Figure 4 and Supplementary Table A1).

#### Safety and Tolerability

The vaccine was well tolerated, with 14 AEs reported in 14 (14.0%) patients at the 3-month visit, all rated as grade 1; 3 (3.0%) had missing data. Of these 14 AEs, 6 were considered related to the immunization and consisted of mild local mucosal irritation. Only 2 (2.0%) patients discontinued treatment due to vaccine-related events. The complete AE profile is presented in Supplementary Table A1.

### PRO Substudy

**Figure 5.**
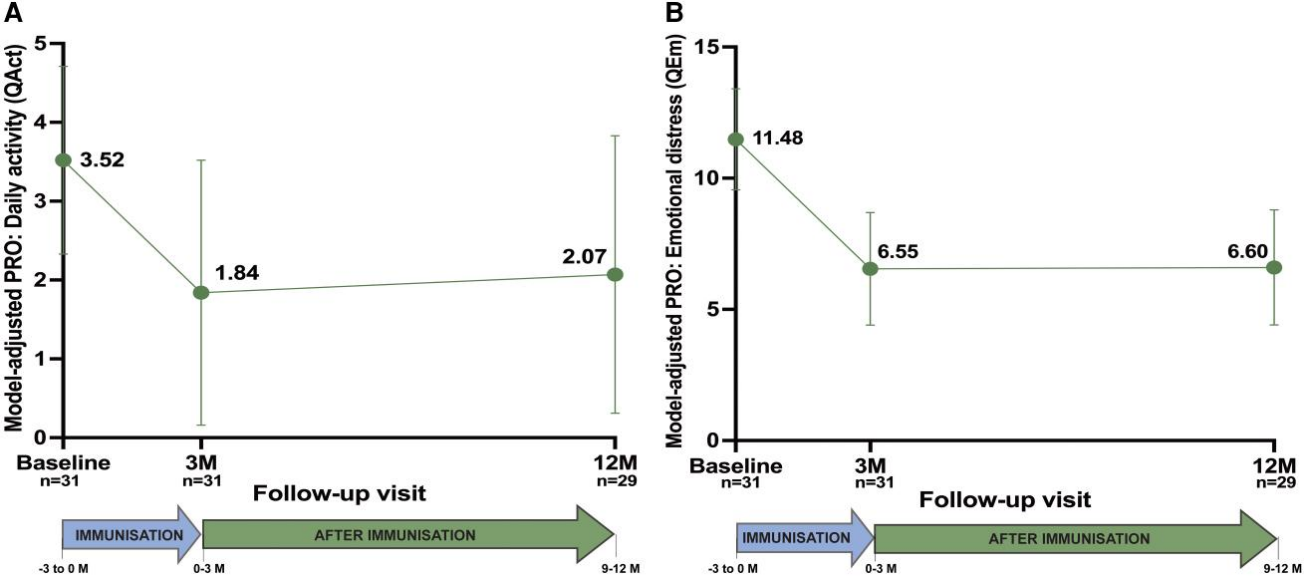
Evolution of patient-reported outcomes (PROs) after individualized immunoprophylaxis. QAct, daily activity domain; QEm, emotional distress domain; M, months. Adjusted mean scores over time obtained from linear mixed-effects models. *A*, Model-adjusted PRO: Daily activity (QAct). *B*, Model-adjusted PRO: Emotional distress (QEm).

Among participants in the PRO substudy, model-adjusted QAct and QEm scores [64] differed from baseline after immunization. A borderline significant (*P* = .050) decrease in QAct scores was evidenced from baseline to the 3-month visit and remained stable (*P* = .100) at 12 months. In contrast, QEm scores were significantly lower at both 3 (*P* < .001) and 12 months (*P* < .001); Figure 5. Corresponding model estimates appear in Supplementary Table A1.

At 12 months, patient satisfaction scores [65, 66] were high, with most respondents reporting values ≥4 on a 5-point scale and nearly all indicating they would choose to undergo autovaccination again (Supplementary Table A1).

## DISCUSSION

This prospective cohort study observed that individualized autovaccination with Uromune® was associated with a lower rUTI episode frequency in a real-world population of older women with a high burden of nmRFs. Nearly half of the patients became UTI-free at 3 months, and recurrence rates remained significantly lower than baseline throughout 12-month follow-up. Negative UCs were more frequent after immunization, with values at 12 months approaching those observed at 3 months, and PROs showed significantly lower scores, particularly in emotional well-being. The vaccine was well tolerated, with only mild AEs reported. Together, these findings suggest that personalized immunoprophylaxis may represent a potential nonantibiotic preventive option for women with rUTIs, even in complex clinical settings. In practice, it may be considered after behavioral measures and correction of reversible risk factors, alongside vaginal estrogen in postmenopausal women and other nAMP options such as methenamine hippurate, particularly when recurrences persist or long-term antibiotic prophylaxis is undesirable.

Our cohort represents a clinically challenging population, characterized by advanced age, multiple nmRFs, and high baseline recurrence rates. In this context, individualized autovaccination was associated with reductions in recurrence that were within the range of those reported for the preformulated MV140 vaccine in younger or less complex populations [50, 52, 53, 58, 59]. Only a few studies have evaluated personalized autovaccines [60–63], generally with smaller samples and heterogeneous methodologies. Our findings therefore provide real-world evidence to an area with limited data. The magnitude of recurrence reduction observed in our cohort also appears broadly consistent with that reported for methenamine hippurate and continuous antibiotic prophylaxis in women with rUTIs, although differences in study design, populations, and outcome definitions preclude direct comparison [23, 31, 32, 63].

The proportion of UTI-free patients (∼40% at both 9 and 12 months) was consistent with the upper range reported in MV140 studies and comparable to rates described for individualized autovaccines to date [60, 62, 63]. Differences in definitions of “UTI-free” status, patient composition, and follow-up periods complicate comparisons, but the overall magnitude of the observed association is in line with previous immunotherapy research.

Bacteriological outcomes were consistent with the observed clinical findings. The proportion of negative UCs increased after treatment, with variability across follow-up visits. Model-adjusted analyses showed fluctuations between visits but indicated sustained differences relative to baseline. When compared with earlier studies, which frequently reported decreasing rates of negative UCs over time [50, 60, 63], our findings suggest a sustained bacteriological response. Negative UCs should not be interpreted as definitive eradication of uropathogens. In rUTI prevention, microbiological improvement may instead reflect reduced colonization or temporary suppression, which may still be clinically meaningful if accompanied by fewer symptomatic episodes.

A potential explanation for the observed clinical and bacteriological results may relate to the individualized nature of our vaccines, which differ from standardized preformulated MV140, and to our protocol for local isolation and purification of uropathogens before vaccine preparation, rather than direct urine shipment to manufacturers as in some previous autovaccine approaches. Although not directly demonstrated for individualized autovaccines, studies of sublingual MV140 support Th1/Th17 and IL-10 immune responses and suggest cross-reactive mucosal immunity, including urinary IgA, against heterologous *E. coli* strains. In our study, closer antigenic matching to each patient's urinary isolates may provide an additional biologically plausible explanation for the observed associations [49, 67].

Importantly, neither age, prior antibiotic prophylaxis, nor cumulative risk-factor burden showed a consistent association with clinical or microbiological outcomes. Although isolated interactions were observed at specific time points, these were inconsistent across follow-up. Given the sample size, these interaction analyses should be considered exploratory. These findings suggest that individualized autovaccination may remain associated with favorable outcomes even in patients with predominantly nmRFs. However, these exploratory analyses may have been underpowered to detect heterogeneity. This potential consistency may be particularly relevant in older women.

The safety profile observed was favorable. Consistent with previous MV140 and autovaccine studies, AEs were mild, localized, and infrequent [50, 52, 53, 58–63]. The low discontinuation rate suggests good tolerability of the sublingual route and supports its potential use in long-term preventive strategies in older or medically complex patients.

HRQoL outcomes observed in the PRO substudy further support the clinical relevance of immunoprophylaxis [64–66]. Emotional distress showed a marked and sustained decrease, and daily activity limitations exhibited a trend toward improvement. PROs have been underreported in previous immunotherapy trials; most relied on generic tools administered at a single follow-up [53, 59, 62]. In contrast, this study applied targeted, domain-specific instruments at 3 time points, reflecting sustained patient-reported benefit over 12 months. High patient satisfaction and willingness to repeat treatment indicate the acceptability of the approach. Given the exploratory nature of the PRO substudy, improved emotional scores may partly reflect perceived disease control or expectation effects.

Although no cost analysis was performed, prior studies have suggested that bacterial immunotherapy may reduce healthcare costs by decreasing rUTI episodes and antibiotic consumption [63, 68]. Given the sustained associations observed in this study, similar cost savings may be plausible, although formal evaluation is needed. Because all rUTI episodes in our study required antibiotic treatment, the observed reduction in recurrences likely also translated into fewer antibiotic courses per patient-year, although this was not prospectively quantified. This potential reduction in antibiotic exposure aligns with global antimicrobial stewardship recommendations to mitigate AMR [26, 27, 69, 70].

The prospective design, real-world enrollment, longitudinal analyses, and 12-month follow-up strengthen the overall interpretation of the results. Nonetheless, several limitations should be acknowledged. First, the observational design without a comparator group limits causal inference and does not preclude residual confounding by indication or patient motivation. Regression toward the mean, behavioral changes after study enrollment, increased clinical follow-up, and attrition bias are potential concerns in recurrent diseases; however, the magnitude and durability of the observed associations, the absence of a consistent modifying effect of baseline risk factors, together with consistent microbiological findings, argue against this as the sole explanation. Second, loss to follow-up, including discontinuations due to AEs, perceived lack of benefit, or initiation of antibiotic prophylaxis, may introduce bias; however, censoring these cases at the time of withdrawal mitigates their influence on longitudinal estimates. Third, the sample size, while adequate for exploratory analyses, limits the precision of subgroup assessments, particularly for PROs, and may introduce selection bias. Fourth, initiation of vaginal estrogen and other concurrent behavioral interventions during follow-up were not systematically recorded, which may represent a potential source of bias. In addition, because UCs were not systematically obtained for each symptomatic episode, some outcome misclassification cannot be excluded. Relationships between these factors, the intervention, and study outcomes are summarized in Supplementary Figure A5. Finally, while the use of locally purified isolates may have enhanced diagnostic accuracy, it also represents a procedural deviation from standard autovaccine preparation in other studies; the reproducibility, scalability and generalizability of this approach beyond older women treated in a specialized Spanish clinic, particularly in younger populations or settings without centralized microbiology support, where individualized manufacturing may be less feasible, warrant further evaluation. Moreover, individualized autovaccines raise regulatory and translational challenges due to their patient-specific composition, despite standardized manufacturing, including product comparability and quality control considerations to ensure scalability and broader clinical adoption. In this context, future studies should include randomized placebo-controlled designs, comparisons with standardized MV140 formulations, and evaluation of antibiotic consumption, cost-effectiveness, and ecological impact.

## CONCLUSIONS

Individualized Uromune® autovaccination was safe and associated with sustained reductions in rUTI recurrences, an increased proportion of negative UCs, and improved patient-reported well-being in a real-world cohort of women with multiple nmRFs. Although encouraging, these observational findings require confirmation in controlled studies to better define the role of personalized immunoprophylaxis in rUTI management.

## Supplementary Material

ofag315_Supplementary_Data

## Supplementary data

Supplementary materials are available at *Open Forum Infectious Diseases* online. Consisting of data provided by the authors to benefit the reader, the posted materials are not copyedited and are the sole responsibility of the authors, so questions or comments should be addressed to the corresponding author.
