# Supplementary material for: Effectiveness of Individualized Autovaccine Immunotherapy in Women With Recurrent Urinary Tract Infections: A Prospective Cohort Study
Source: Open Forum Infect Dis. 2026 May 21;13(6):ofag315. doi: 10.1093/ofid/ofag315 (PMC13233108; doi:10.1093/ofid/ofag315)
Supplement: ofag315_Supplementary_Data [file ofag315_supplementary_data.docx]

**Appendix - Effectiveness of Individualized Autovaccine Immunotherapy in Women With Recurrent Urinary Tract Infections: A Prospective Cohort Study**

Diaz-Brito, V. *et al*.

**Figure A1.** Heatmap of cumulative risk factors among women with recurrent UTI at baseline visit.


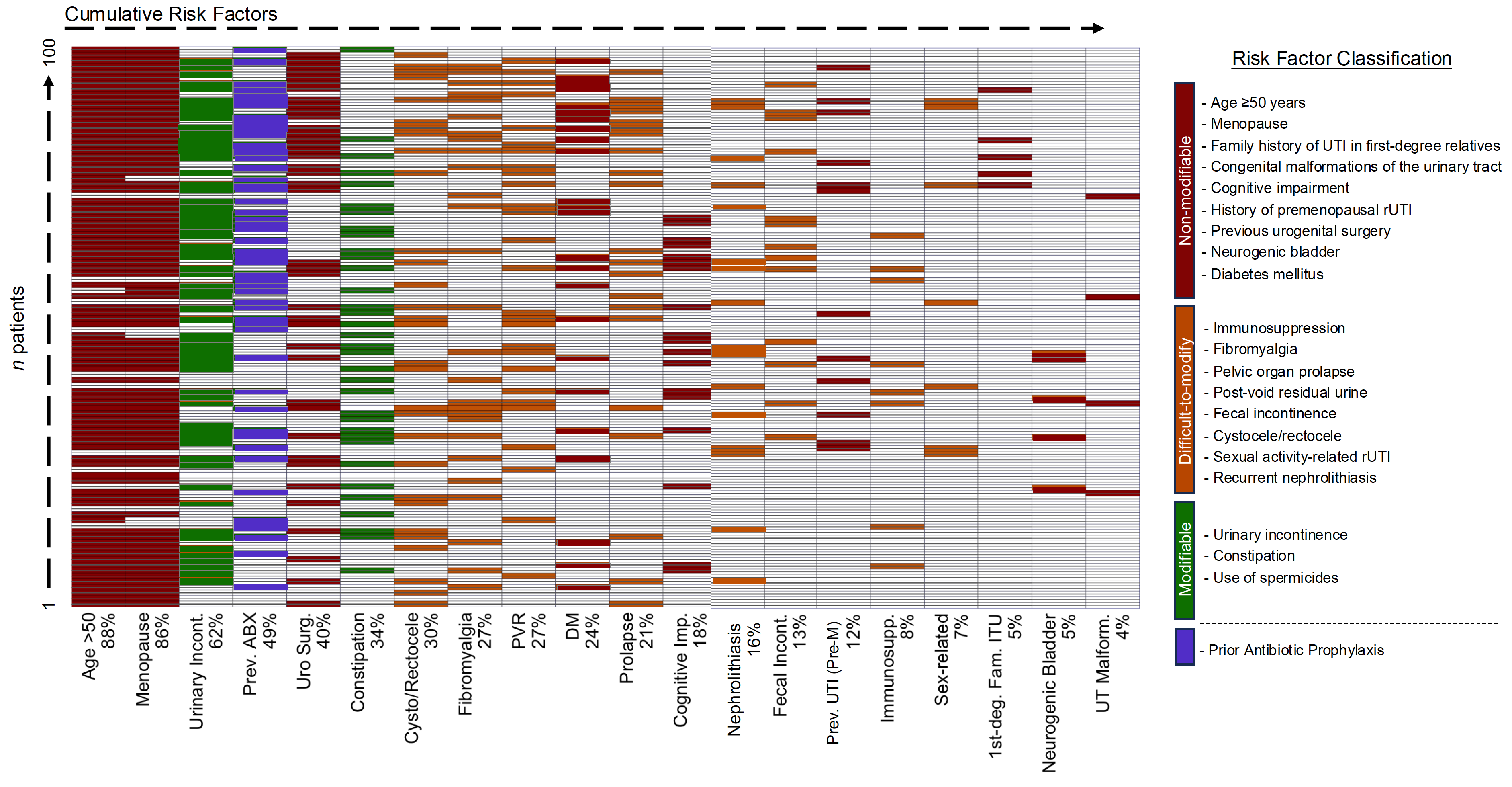


Urinary Incont: urinary incontinence, Prev. ABX: previous antibiotic prophylaxis, PVR: post-void residual urine, DM: diabetes mellitus, Prolapse: pelvic organ prolapse, Cognitive Imp: cognitive impairment, Fecal incont: fecal incontinence, Prev. UTI (Pre-M): history of premenopausal recurrent urinary tract infection (UTI), Immunosupp: immunosuppression, Sex-related: sexual activity-related recurrent UTI, 1st-deg. Fam. UTI: family history of UTI in first-degree relatives, UT Malform: congenital mal-formations of the urinary tract.

Each column represents one risk factor and each row represents an individual patient (n = 100). Colored cells indicate the presence of the corresponding risk factor, grouped into non-modifiable, difficult-to-modify, and modifiable categories. Prior antibiotic prophylaxis is shown separately. Percentages below each factor indicate prevalence in the cohort. Risk-factor classification is displayed in the legend on the right.

**Figure A2.** Distribution of all microorganisms used for autovaccine formulation.


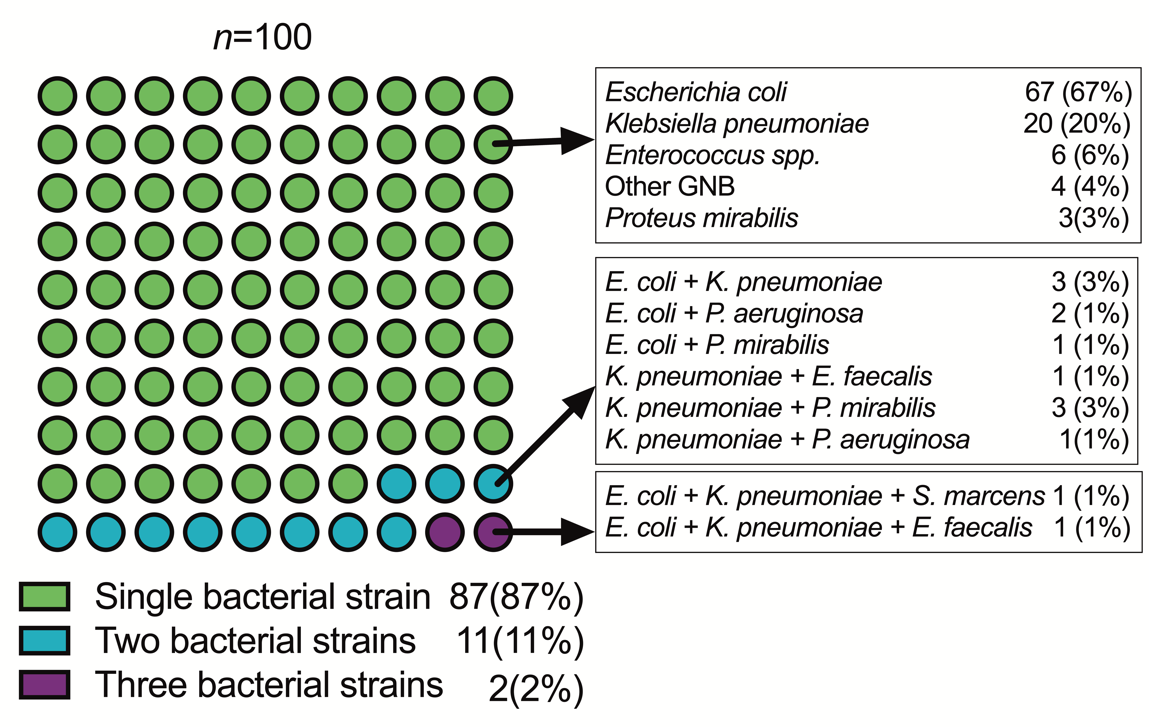


*E. coli: Escherichia coli, K. pneumoniae: Klebsiella pneumoniae, P. aeruginosa: Pseudomonas aeruginosa, P. mirabilis: Proteus mirabilis, E. faecalis: Enterococcus faecalis, S. marcens: Serratia marcescens*, Other GNB: other Gram-negative bacilli.

Each dot represents one bacterial species isolated from a patient’s own urine culture (multiple dots may correspond to different isolates from the same culture) and incorporated into her personalized vaccine. Green dots correspond to patients whose vaccine contained a single bacterial species, blue dots to vaccines comprising two species, and purple dots to those including three species.

**Figure A3.** Impact of individualized prophylactic autovaccine on rUTI episodes over time.


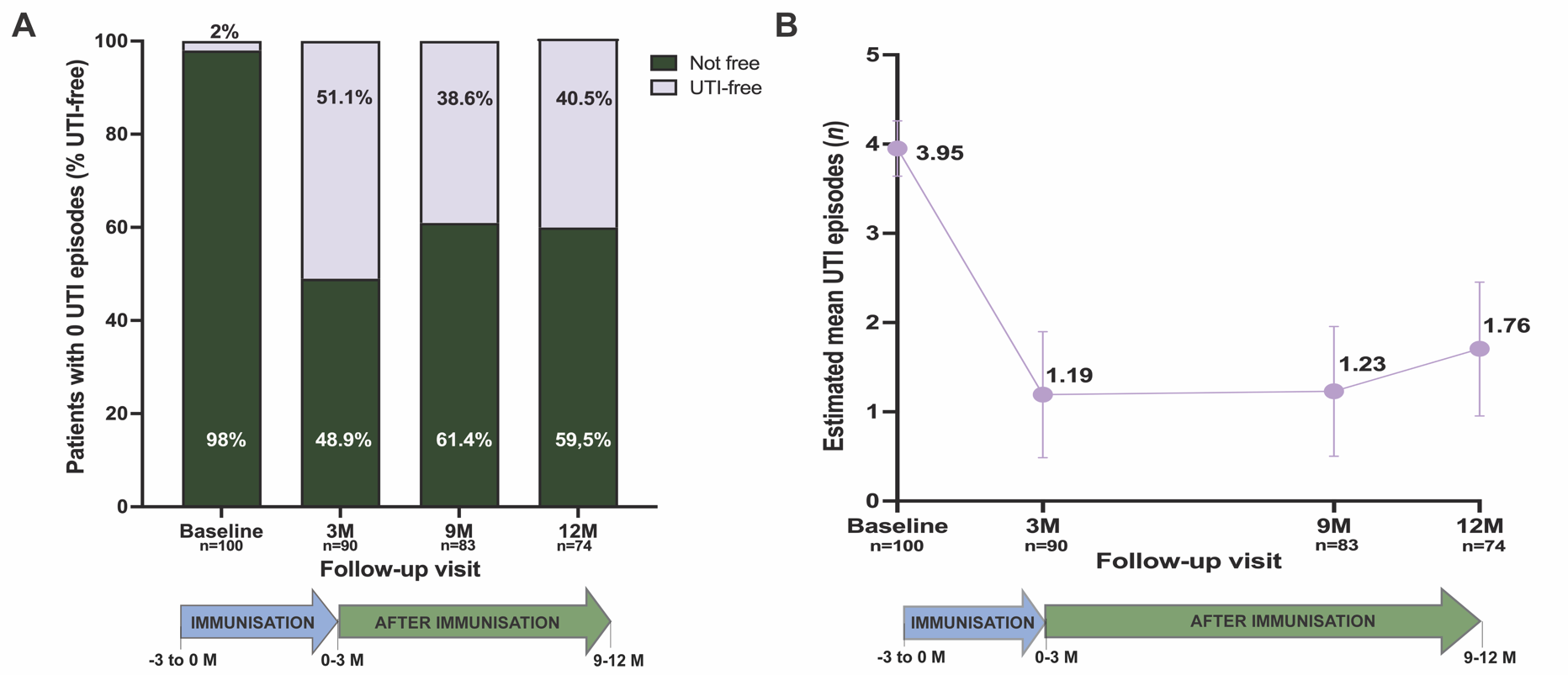


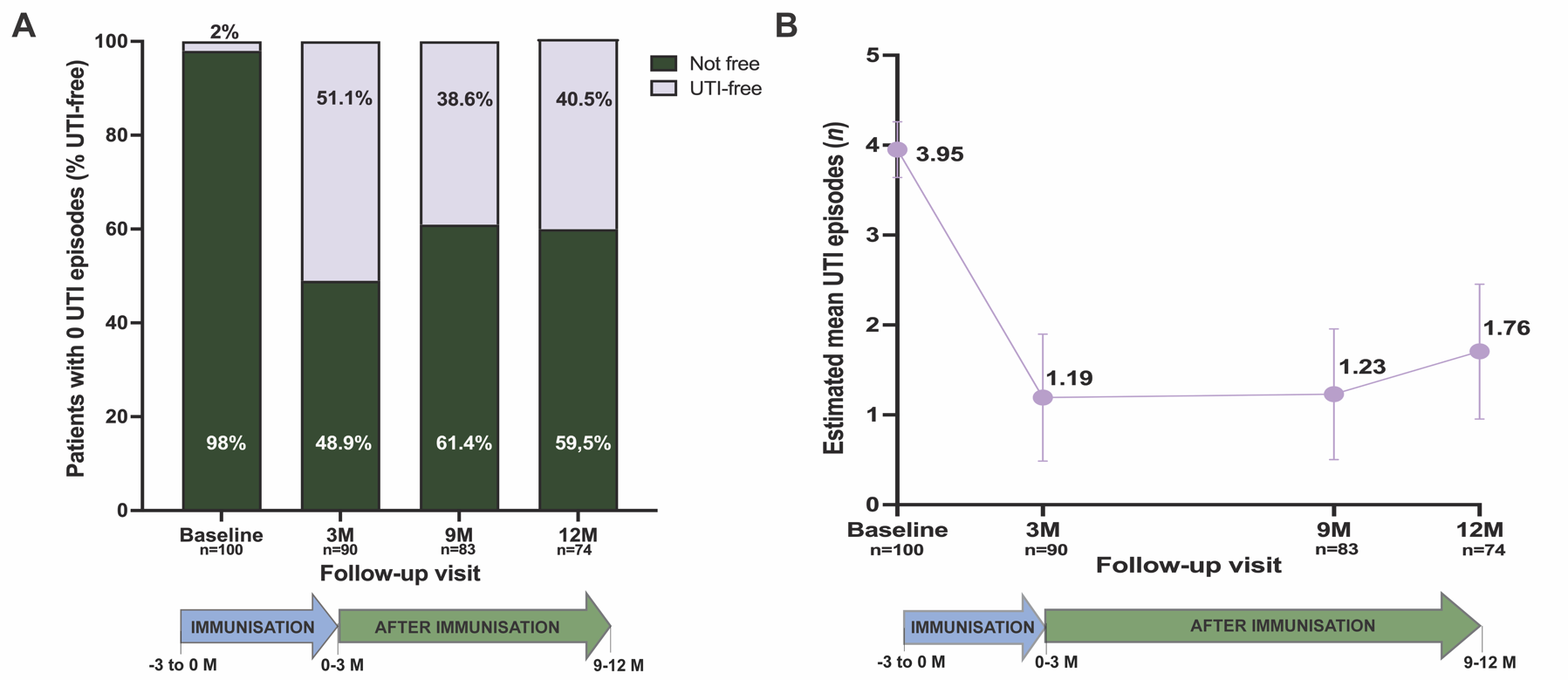


UTI: Urinary Tract Infection, M: months.

(a) Proportion of UTI-free patients at each visit. Stacked bars show the percentage of patients with 0 UTI episodes (UTI-free) and ≥1 episode at baseline and during follow-up. Differences versus baseline were evaluated using McNemar's test.

(b) Longitudinal model-estimated mean number of UTI episodes. Adjusted mean UTI episode counts over time, obtained from a population-averaged longitudinal model.

**Figure A4.** Bacterial strains included in autovaccines and uropathogens isolated during the 12-month follow-up.


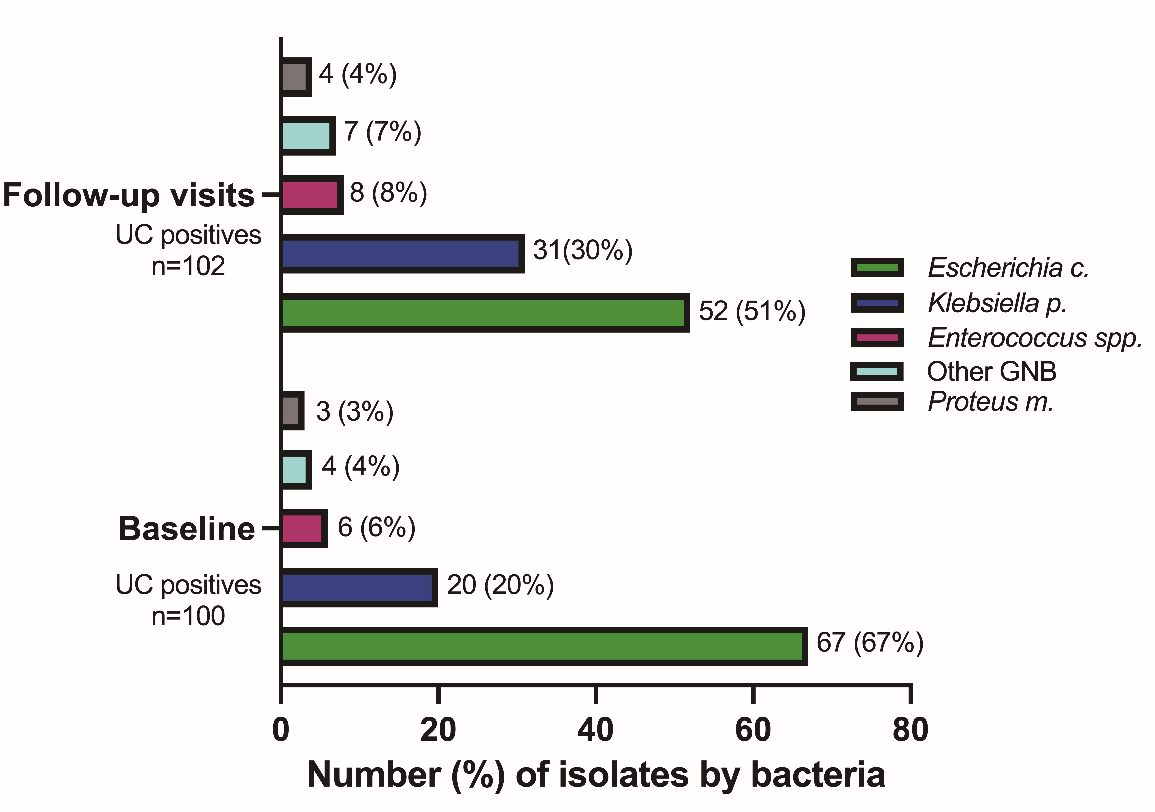


*Escherichia c.: Escherichia coli, Klebsiella p.: Klebsiella pneumoniae, Enterococcus spp.: Enterococcus species,* Other GNB: other Gram-negative bacilli*, Proteus m.: Proteus mirabilis.*

Baseline isolates correspond to the uropathogens purified for individualized autovaccine preparation, whereas follow-up isolates represent all pathogens recovered from positive urine cultures throughout the 12-month study period. Bars represent the absolute number and the percentage of isolates corresponding to each bacterial species.

**Figure A5.** Conceptual framework of baseline risk factors, potential confounders, individualized autovaccine intervention, and study outcomes**.**

Baseline risk factors, recurrence burden, behavioral changes during follow-up, vaginal estrogen use, follow-up intensity, and selective dropout of non-responders may influence both the likelihood of response to individualized autovaccine treatment and the observed study outcomes, including rUTI episodes, bacteriological clearance, and patient-reported outcomes (PRO) improvement.

**Table A1.** Baseline risk factors, age, and prior antibiotic prophylaxis, and their effect on longitudinal clinical and bacteriological outcomes.

|  | **Baseline** (*n*=100) | | **3M** (*n*=90) | | **9M** (*n*=83) | | **12M** (*n*=74) | |
| --- | --- | --- | --- | --- | --- | --- | --- | --- |
|  | **Unadjusted model** | | **Adjusted longitudinal models** | | | | | |
|  | **Descriptive** | **Association** | **UTIe ef.**  **(*p*)** | **UC neg ef.**  **(*p*)** | **UTIe ef.**  **(*p*)** | **UC neg ef.**  **(*p*)** | **UTIe ef.**  **(*p*)** | **UC neg ef.**  **(*p*)** |
| Age m (IQR) years | 73 (61-80) | = (*p*=0.80) | 0.8 (=) | 0.19 (=) | 0.91 (=) | **<0.01 (↓)** | 0.94 (=) | 0.09 (=) |
| Prev. ABX prophylaxis *n*(%) | 49 (49%) | = (*p*=0.67) | 0.13 (=) | 0.25 (=) | 0.11 (=) | 0.56 (=) | **0.02 (↑)** | 0.85 (=) |
| **Risk factors** | | |  |  |  |  |  |  |
| Total risk factors p/p m (IQR) | 4 (3-6) | = (*p*=0.21) | 0.18 (=) | 0.16 (=) | **0.02 (↑)** | 0.79 (=) | **0.01 (↑)** | 0.81 (=) |
| nmRF p/p m (IQR) | 3 (2-4) | **↓ (*p=*0.04)** | 0.98 (=) | 0.44 (=) | 0.13 (=) | 0.31 (=) | 0.22 (=) | 0.27 (=) |
| Age>50 years *n*(%) | 88 (88%) | = (*p*=0.71) | 0.71 (=) | 0.87 (=) | **0.01 (↑)** | **0.03 (↓)** | 0.58 (=) | 0.29 (=) |
| Menopause *n*(%) | 86 (86%) | = (*p*=0.5) | 0.61 (=) | 0.83 (=) | 0.14 (=) | 0.09 (=) | **0.03 (↑)** | 0.37 (=) |
| Urogenital surgery *n*(%) | 40 (40%) | **↓ (*p*<0.01)** | **0.03 (↑)** | 0.11 (=) | **<0.01 (↑)** | 0.42 (=) | **<0.01 (↑)** | 0.12 (=) |
| Diabetes Mellitus *n*(%) | 24 (24%) | = (*p*=0.31) | **0.04 (↑)** | 0.52 (=) | 0.14 (=) | 0.68 (=) | 0.12 (=) | 0.40 (=) |
| Cognitive impairment *n*(%) | 18 (18%) | = (*p*=0.23) | 0.57 (=) | 0.05 (=) | 0.45 (=) | 0.71 (=) | 0.08 (=) | 0.43 (=) |
| Prev. UTI (Pre-M) *n*(%) | 12 (12%) | = (*p*=0.31) | 0.51 (=) | 0.18 (=) | **0.01 (↑)** | 0.51 (=) | **<0.01 (↑)** | 0.85 (=) |
| Neurogenic bladder *n*(%) | 5 (5%) | = (*p*=0.48) | 0.44 (=) | NA | 0.49 (=) | NA | 0.16 (=) | NA |
| 1st-deg. Fam. UTI *n*(%) | 5 (5%) | = (*p*=0.16) | 0.11 (=) | NA | **0.04 (↑)** | NA | 0.15 (=) | NA |
| UT. malformation *n*(%) | 4 (4%) | = (*p*=0.17) | 0.45 (=) | NA | 0.18 (=) | NA | 0.29 (=) | NA |
| Diffi. to modify RF p/p m (IQR) | 1 (1-2) | = (*p*=0.34) | 0.66 (=) | **0.02 (↑)** | 0.92 (=) | 0.35 (=) | 0.73 (=) | 0.49 (=) |
| Cystocele/Rectocele *n*(%) | 30 (30%) | = (*p*=0.88) | 0.64 (=) | 0.47 (=) | 0.43 (=) | 0.74 (=) | **<0.02 (↑)** | 0.22 (=) |
| Fibromyalgia | 27 (27%) | = (*p*=0.39) | 0.86 (=) | **0.03 (↓)** | 0.97 (=) | 0.68 (=) | 0.28 (=) | 0.77 (=) |
| Post-void residual urine *n*(%) | 27 (27%) | = (*p*=0.43) | 0.56 (=) | NA | 0.43 (=) | NA | 0.79 (=) | NA |
| Pelvic organ prolapse *n*(%) | 21 (21%) | = (*p*=0.66) | 0.51 (=) | 0.11 (=) | 0.24 (=) | 0.39 (=) | 0.14 (=) | 0.77 (=) |
| Recurrent nephrolithiasis *n*(%) | 16 (16%) | = (*p*=0.22) | 0.34 (=) | NA | 0.51 (=) | NA | 0.41 (=) | NA |
| Fecal incontinence *n*(%) | 13 (13%) | = (*p*=0.64) | 0.99 (=) | 0.58 (=) | 0.79 (=) | 0.89 (=) | 0.71 (=) | 0.20 (=) |
| Immunosuppression *n*(%) | 8 (8%) | = (*p*=0.30) | 0.54 (=) | NA | 0.09 (=) | NA | 0.1 (=) | NA |
| Sex-related *n*(%) | 7 (7%) | = (*p*=0.32) | 0.25 (=) | 0.12 (=) | 0.85 (=) | 0.35 (=) | 0.92 (=) | 0.91 (=) |
| Modifiable RF p/p m (IQR) | 1 (1-2) | = (*p*=0.45) | 0.85 (=) | 0.81 (=) | 0.10 (=) | 0.45 (=) | 0.46 (=) | 0.47 (=) |
| Urinary incontinence *n*(%) | 62 (62%) | = (*p*=0.99) | 0.99 (=) | 0.37 (=) | 0.26 (=) | 0.17 (=) | 0.47 (=) | 0.16 (=) |
| Constipation *n*(%) | 34 (34%) | = (*p*=0.73) | 0.86 (=) | 0.55 (=) | 0.78 (=) | 0.78 (=) | 0.73 (=) | 0.68 (=) |
| Use of spermicides *n*(%) | 0 (0%) | NA | NA | NA | NA | NA | NA | NA |

p/p: per patient, m: median, IQR: interquartile range, NA: not available, M: months, RF: risk factor, nmRF: non-modifiable risk factor, Diffi. to modify: difficult to modify, UTIe ef: urinary tract infection episodes effect, UC neg ef.: urine culture negative effect, Prev. ABX prophylaxis: previous antibiotic prophylaxis, Prev. UTI (Pre-M): history of premenopausal recurrent urinary tract infection, 1st-deg. Fam. UTI: family history of UTI in first-degree relatives, UT. Malform: congenital malformations of the urinary tract, Sex-related: sexual activity-related recurrent UTI.

Baseline association reflects results from unadjusted analyses at baseline (UTI episodes only). For UTI episodes, baseline was included in the adjusted longitudinal mixed-effects models. Because all baseline urine cultures were considered positive, baseline was excluded from longitudinal modeling for UC negativity. For follow-up visits (3M, 9M, 12M), p-values reflect time-by-factor interaction effects from adjusted longitudinal models. Symbols indicate the direction of the effect when p < 0.05: (=) no differential effect; (↑) worse evolution; (↓) better evolution.

**Table A2.** Adverse events assessed at the 3-month visit (n=100).

| **Overall adverse events** |  |  |  | **n (%)** |
| --- | --- | --- | --- | --- |
| No adverse events reported |  |  |  | 83 (83.0) |
| Adverse events reported |  |  |  | 14 (14.0) |
| Not evaluated |  |  |  | 3 (3.0) |
| **AEs related to vaccination** | | |  | |
| **Type of AE** | **Grade** | **Category** | **Discontinuation, n (%)** | **Total, n (%)** |
| Mucosal irritation, n (%) | 1 | Local | 2 (2.0) | 6 (6.0) |
|  |  |  | Total AEs, n (%) | 6 (42.9) |
| **AEs not related to vaccination** | | | | |
| Headache | 1 | Systemic | 0 | 1 (1.0) |
| Insomnia | 1 | Systemic | 1 | 1 (1.0) |
| Rash | 1 | Systemic | 0 | 1 (1.0) |
| Constipation | 1 | Systemic | 0 | 1 (1.0) |
| Dizziness | 2 | Systemic | 2 | 2 (2.0) |
| Gastroesophageal reflux | 1 | Systemic | 1 | 1 (1.0) |
| Difficulty concentrating | 1 | Systemic | 1 | 1 (1.0) |
|  |  |  | Total AEs (%) | 8 (57.1) |

AEs: adverse events. All events are reported per patient, as no participant experienced more than one AE.

**Table A3.** PRO Substudy: Adjusted Model Estimates for QAct and QEm Scores Over Time.

| **Domain** | Timepoint | n | Model-Estimated Mean (95% CI) | Change vs Baseline (p) |
| --- | --- | --- | --- | --- |
| **QAct** | Baseline | 31 | 3.52 (2.33 to 4.71) | - |
|  | 3M | 31 | 1.84 (−1.03 to 4.72) | −1.68 (0.055) |
|  | 12M | 29 | 2.07 (−0.83 to 4.98) | −1.45 (0.103) |
|  |  |  |  |  |
| **QEm** | Baseline | 31 | 11.48 (9.56 to 13.41) | - |
|  | 3M | 31 | 6.55 (2.48 to 10.62) | −4.94 (<0.001) |
|  | 12M | 29 | 6.60 (2.47 to 10.70) | −4.89 (<0.001) |

PRO: patient-reported outcomes, QAct: daily activity domain, QEm: emotional distress domain; M: months, Chg. Vs Baseline: Change vs Baseline visit, p: p-value.

**Table A4.** PRO Substudy: Patient Satisfaction at 12-month visit (n=29).

| **Satisfaction score** | **n (%)** |
| --- | --- |
| 1 | 2 (6.9) |
| 2 | 3 (10.3) |
| 3 | 5 (17.2) |
| 4 | 8 (27.6) |
| 5 | 11 (37.9) |
| **Willingness to repeat** | **n (%)** |
| Yes | 28 (96.7) |
| No | 1 (3.3) |

Satisfaction was assessed using a 5-point scale derived from the TSQM framework, where 1 indicates very low satisfaction and 5 indicates maximal treatment satisfaction.
